# The Role of Inflammatory Biomarkers and Microbiological Profile in Clinical Decision-Making for Antibiotic Therapy in Pediatric Patients: A Retrospective Study

**DOI:** 10.3390/antibiotics15101020

**Published:** 2026-10-14

**Authors:** Fitim Bexhet Alidema, Arieta Hasani Alidema, Njomza Lajçi Berisha

**Affiliations:** 1 Department of Pharmacology, Faculty of Pharmacy, UBT College, 1000 Prishtina, Kosovo; 2 Department of Nursing, Faculty of Medical, UBT College, 1000 Prishtina, Kosovo; 3 Department of Pharmacology, Faculty of Pharmacy, University of Prishtina, 1000 Prishtina, Kosovo

**Keywords:** antimicrobial stewardship, pediatric infections, antibiotic prescribing, inflammatory biomarkers, C-reactive protein, procalcitonin, microbiological testing, antibiotic decision-making, diagnostic stewardship

## Abstract

Background: Distinguishing bacterial from non-bacterial infections remains challenging in pediatric practice. This study evaluated the relationship between inflammatory biomarkers, microbiological evidence, and antibiotic prescribing, with particular emphasis on agreement between treatment decisions and a study-specific bacterial evidence classification. Methods: This retrospective observational study included 2688 pediatric patients evaluated between January 2023 and March 2026. A nonvalidated, study-specific Bacterial Evidence Score (BES; 0–6 points) combined fever, C-reactive protein, white blood cell count, absolute neutrophil count, procalcitonin, and positive bacterial culture. The primary outcome was disagreement between antibiotic prescribing and the study-defined BES threshold (≥2), rather than independently confirmed inappropriate prescribing. Multivariable logistic regression was used to evaluate factors associated with antibiotic prescribing and BES-based decision disagreement. Results: Antibiotics were prescribed to 1974 patients (73.4%), of whom 1391 (70.5%) received agents classified as broad-spectrum. Microbiological testing was performed in 913 patients (34.0%), yielding 362 positive cultures. Antibiotic prescribing increased overall across BES levels, from 36.9% at score 0 to 98.1% at score 6 (adjusted odds ratio per point, 1.28; 95% confidence interval, 1.17–1.41). Under the study-specific BES ≥ 2 classification, 825 decisions (30.7%) disagreed with the scoring rule; this proportion does not represent the prevalence of clinically inappropriate prescribing. Among culture-positive patients, 167 (46.1%) received pathogen-directed antibiotic therapy supported by microbiological findings, and 91 (25.1%) underwent microbiology-guided treatment modification. Clinical resolution or improvement was documented in 2224 patients (82.7%). Conclusions: Antibiotic prescribing was associated with cumulative inflammatory and microbiological evidence. However, the BES-based classification was exploratory and sensitive to the selected threshold, while microbiological findings were not necessarily available at initial prescribing. These findings support further evaluation of integrated diagnostic and antimicrobial stewardship approaches, with prospective validation required before clinical application.

## 1. Introduction

Antimicrobial resistance (AMR) represents one of the major challenges to contemporary healthcare, with inappropriate and excessive antibiotic exposure contributing substantially to the selection and dissemination of resistant bacterial pathogens. A comprehensive global analysis estimated that bacterial AMR was attributable to approximately 1.27 million deaths and associated with 4.95 million deaths worldwide in 2019, emphasizing the need for more precise and evidence-based antimicrobial use across all age groups [1]. Pediatric populations are particularly relevant to antimicrobial stewardship because antibiotics are frequently prescribed during childhood. Outpatient pediatric care accounts for a substantial proportion of antibiotic exposure, and considerable variability in outpatient antibiotic prescribing has been documented [2].

Antimicrobial stewardship in pediatrics extends beyond reducing the overall number of prescriptions and requires optimization of the indication, antimicrobial agent, dose, route, and duration of treatment. Contemporary pediatric stewardship policies therefore emphasize that antibiotics should be prescribed only when necessary and, when indicated, selected and administered in a manner that optimizes clinical outcomes while minimizing the unintended consequences of antibiotic use [3]. Systematic evidence from pediatric emergency departments and primary care has demonstrated that antimicrobial stewardship interventions can improve antibiotic prescribing and adherence to clinical guidelines, although considerable heterogeneity exists in the types of interventions implemented and outcomes evaluated [4]. More recently, antimicrobial stewardship has increasingly been integrated with diagnostic stewardship, recognizing their complementary roles in optimizing antimicrobial use and improving diagnostic accuracy to guide appropriate treatment decisions [5].

Clinical uncertainty remains an important determinant of antibiotic prescribing in children. Commonly used biomarkers, including white blood cell count, C-reactive protein, and procalcitonin, have limitations in distinguishing serious bacterial infections from other causes of fever. Such diagnostic uncertainty may contribute to antibiotic overprescribing in pediatric emergency care [6]. Inflammatory biomarkers may provide additional objective information during the assessment of suspected bacterial infection. C-reactive protein (CRP) and procalcitonin (PCT) are among the biomarkers evaluated for this purpose; however, their discriminatory performance is not definitive. In children presenting with fever and features of possible sepsis or meningitis, CRP and PCT showed identical moderate discriminatory performance for serious bacterial infection, with an area under the curve of 0.70 for each biomarker [7].

Procalcitonin has received particular attention as a biomarker capable of supporting diagnostic decision-making; however, its diagnostic performance is not sufficiently precise to be interpreted in isolation. A systematic review and meta-analysis of 25 studies involving 2864 children demonstrated moderate diagnostic accuracy of PCT for bacterial pneumonia and recommended that it be used in conjunction with other clinical and diagnostic findings [8]. Evidence from randomized pediatric studies suggests that PCT-guided strategies may reduce the duration of antibiotic therapy and antibiotic-related adverse events, while no significant differences have been demonstrated in antibiotic prescription rates, length of hospital stay, hospital readmission, or mortality [9]. Similarly, observational evidence in children with community-acquired pneumonia has demonstrated discordance between objective biomarker-based estimates of bacterial etiology and empirical antibiotic decisions. In a prospective cohort in which PCT results were blinded to clinicians, PCT values were not associated with either antibiotic initiation or the selection of narrow- versus broad-spectrum empirical therapy, highlighting important opportunities for antimicrobial stewardship [10]. These findings suggest that individual biomarkers are most informative when integrated with other indicators of bacterial disease rather than interpreted through isolated thresholds.

Microbiological testing provides a complementary dimension to inflammatory biomarkers by enabling pathogen identification and, when antimicrobial susceptibility information is available, supporting the refinement of empirical therapy. In pediatric bloodstream infections, rapid diagnostic testing may facilitate earlier optimization of antimicrobial treatment, particularly when implemented in conjunction with an effective antimicrobial stewardship program [11]. Accordingly, contemporary pediatric antimicrobial stewardship extends beyond reducing antibiotic use and emphasizes the implementation of evidence-based, best-practice approaches to antibiotic therapy. Multinational expert consensus has established specific recommendations for antimicrobial stewardship programs in hospital settings to improve the quality of antibiotic therapy for children [12]. These principles are particularly important in the context of multidrug-resistant organisms and other resistance phenotypes, for which empirical therapy may be inadequate or unnecessarily broad.

Despite growing evidence supporting pediatric antimicrobial stewardship, important gaps remain regarding the effectiveness of stewardship strategies across different healthcare settings. Evidence from low- and middle-income countries remains comparatively limited, and substantial heterogeneity among available studies restricts conclusions regarding the most effective approaches to pediatric antimicrobial stewardship [13]. Recent evaluations of pediatric antimicrobial and diagnostic stewardship have demonstrated substantial heterogeneity in intervention types, outcomes, and metrics used to assess stewardship programs. This variability limits comparisons across studies and highlights the need for harmonized outcome measures to evaluate and inform pediatric antimicrobial stewardship strategies [14].

Therefore, rather than evaluating inflammatory biomarkers or microbiological findings in isolation, an integrated assessment of the cumulative evidence supporting bacterial infection may provide a more clinically meaningful framework for studying antibiotic decision-making. The present study has evaluated 2688 pediatric patients using demographic, clinical, inflammatory, microbiological, resistance, and pharmacotherapeutic information collected from January 2023 to March 2026. The primary objective has been to investigate the relationship between inflammatory biomarkers, microbiological evidence, and concordance of antibiotic decision-making. In addition, the study has evaluated antibiotic initiation, empirical versus targeted treatment, antimicrobial spectrum, microbiology-guided treatment modification, culture–antibiotic alignment, documented clinical outcomes, and early reassessment. By integrating multiple dimensions of bacterial evidence within a single real-world pediatric cohort, this study provides a clinically applicable antimicrobial stewardship perspective on how diagnostic information is translated into antibiotic treatment decisions.

## 2. Materials and Methods

### 2.1. Study Design and Setting

This retrospective observational analytical study was conducted at the Pediatric Ward, General Hospital of Gjilan, Kosovo. Clinical, laboratory, microbiological, and pharmacotherapeutic data were retrospectively extracted from existing medical records for pediatric patients evaluated between January 2023 and March 2026. The study was designed to investigate the relationship between inflammatory biomarkers, microbiological evidence, and antibiotic prescribing decisions, with the primary objective of assessing agreement between antibiotic initiation or withholding and a study-specific, nonvalidated Bacterial Evidence Score (BES)-based classification.

Secondary and exploratory objectives included evaluating antibiotic selection, antimicrobial spectrum, empirical or targeted treatment, timing of antibiotic initiation, pre-antibiotic microbiological testing, treatment modification following microbiological results, associations between individual bacterial evidence indicators and antibiotic prescribing, documented clinical outcomes, and early reassessment. The study involved no prospective patient recruitment or intervention in clinical management. The final analytical cohort comprised 2688 pediatric patients, with one structured record containing 55 predefined variables for each patient.

### 2.2. Study Population and Case Selection

Pediatric patients whose medical records provided sufficient information to evaluate the primary diagnosis, clinical characteristics, inflammatory parameters, and antibiotic treatment decision were included. Patient selection was based on the eligibility criteria established for the study and the completeness of the available clinical documentation. Data were extracted retrospectively from existing medical records using a structured study-specific data collection instrument. Records were selected according to eligibility and documentation completeness rather than through consecutive sampling of all pediatric presentations during the study period. Eligible records represented acute infectious conditions or clinical suspicion of infection for which sufficient information was available to characterize therapeutic management. When microbiological investigations had been clinically indicated and performed, specimen type, culture results, identified pathogens, and antimicrobial susceptibility testing were also recorded. Records with insufficient information to determine the primary diagnosis, relevant clinical and inflammatory characteristics, or antibiotic treatment decision were excluded, as were duplicate records. For patients with more than one documented clinical encounter, the episode with sufficiently complete information to meet the study eligibility criteria was selected as the index episode. Only one eligible episode per patient was retained in the analytical dataset to avoid duplicate patient-level observations.

### 2.3. Demographic and Clinical Characteristics

For each patient, the index date, calendar year, age in months and years, age group, sex, body weight, care setting, primary diagnosis, infection site, and duration of symptoms were recorded. The initial clinical assessment included maximum body temperature, presence of fever ≥ 38 °C, heart rate, respiratory rate, and peripheral oxygen saturation. Primary diagnoses were categorized according to the diagnostic information documented in the medical records and the predefined categories of the study database, including viral upper respiratory infection, acute otitis media, urinary tract infection, bacterial gastroenteritis, sepsis or suspected invasive infection, streptococcal pharyngotonsillitis, community-acquired pneumonia, acute bacterial sinusitis, and skin and soft-tissue infection. These categories reflected the recorded clinical diagnoses and were not independently validated through a standardized diagnostic adjudication procedure. Primary diagnosis and care setting were considered clinically relevant characteristics in analyses of antibiotic decision-making because the pretest probability of bacterial infection and the indication for antimicrobial treatment may differ across diagnostic categories and clinical settings.

### 2.4. Inflammatory Biomarkers and Hematological Parameters

The inflammatory profile was evaluated using C-reactive protein (CRP), procalcitonin (PCT), total white blood cell count (WBC), absolute neutrophil count (ANC), neutrophil percentage, lymphocyte percentage, and erythrocyte sedimentation rate (ESR). These parameters were interpreted together with body temperature and the overall clinical presentation rather than considering any single biomarker as definitive evidence of bacterial etiology. Numerical biomarker values were retained and analyzed as continuous variables where appropriate to preserve biological information and minimize information loss resulting from unnecessary categorization. Predefined CRP categories available in the database were retained for clinically relevant descriptive and secondary analyses. Separately, CRP, WBC, ANC, and PCT were dichotomized using study-specific thresholds for construction of the Bacterial Evidence Score (BES), as described in Section 2.9. These thresholds were used for exploratory analytical classification and were not considered independently validated diagnostic criteria for bacterial infection.

### 2.5. Microbiological Assessment

For each patient, whether microbiological testing had been requested was recorded. When testing was performed, specimen type, Gram-stain result, culture result, identified pathogen, and polymicrobial growth were documented. Whether the microbiological specimen was obtained before antibiotic initiation was also recorded. Culture results were classified as positive or negative only when microbiological testing had actually been performed. Patients without microbiological testing were retained as a separate category and were not classified as having a negative culture, thereby avoiding misclassification of the absence of testing as the absence of microbiological evidence of bacterial infection.

For construction of the study-specific Bacterial Evidence Score (BES), a positive bacterial culture contributed one point, whereas a documented negative culture or absence of microbiological testing contributed zero points. However, patients with negative cultures and those without microbiological testing remained distinguishable in the analytical dataset, and these two categories were not considered clinically equivalent.

The timing of microbiological specimen collection was distinguished from the availability of culture and antimicrobial susceptibility results. Because the exact time at which individual microbiological results became available was not consistently recorded, it could not be established whether positive culture findings had been available when the initial antibiotic decision was made. Consequently, the microbiological component of the complete BES reflected evidence documented during the index clinical episode rather than necessarily representing information available at initial prescribing.

To examine the influence of microbiological result availability and selective testing on the BES-based classification, a sensitivity analysis was also conducted using a five-component score that excluded bacterial culture and retained the clinical and inflammatory indicators.

### 2.6. Antimicrobial Susceptibility and Resistance Profile

Among patients with relevant microbiological data, the availability of antimicrobial susceptibility testing was recorded, together with clinically relevant antimicrobial resistance phenotypes. Multidrug resistance (MDR), extended-spectrum beta-lactamase (ESBL) production, and methicillin-resistant *Staphylococcus aureus* (MRSA) were recorded according to the resistance phenotypes documented in the available microbiological and antimicrobial susceptibility reports; no retrospective reclassification of resistance phenotypes was performed for the present analysis. These variables were used to characterize the antimicrobial resistance profile and its relationship with antibiotic selection, broad-spectrum therapy, transition from empirical to targeted treatment, and treatment modification following microbiological results. Resistance phenotypes were interpreted only among patients for whom the corresponding microbiological and antimicrobial susceptibility information was available.

Antimicrobial susceptibility testing was not assumed to have been performed in patients without an available antibiogram, and the absence of susceptibility data was not interpreted as evidence of antimicrobial susceptibility or resistance. The availability of an antibiogram was distinguished from its documented use in antibiotic selection or subsequent treatment modification. Accordingly, microbiologically guided treatment was assessed using the recorded relationship between pathogen identification, susceptibility findings, and therapeutic decisions rather than the presence of susceptibility results alone.

### 2.7. Antibiotic Exposure and Pharmacotherapeutic Characteristics

For each patient, antibiotic prescribing during the index clinical episode was recorded. Among antibiotic-treated patients, the individual antimicrobial agent, pharmacological class, route of administration, antimicrobial spectrum, empirical or targeted treatment status, timing of antibiotic initiation, dose expressed as mg/kg/day, daily dosing frequency, and planned treatment duration were documented. Fourteen individual antibiotics were represented in the database: amoxicillin, amoxicillin/clavulanate, ampicillin, penicillin V, cephalexin, cefuroxime, ceftriaxone, cefotaxime, azithromycin, clarithromycin, clindamycin, gentamicin, trimethoprim-sulfamethoxazole, and vancomycin.

Antibiotic treatment was classified using a study-specific operational spectrum classification based on the antimicrobial spectrum of the prescribed agent. Amoxicillin, ampicillin, penicillin V, and cephalexin were classified as narrow-spectrum agents, whereas amoxicillin/clavulanate, cefuroxime, ceftriaxone, cefotaxime, azithromycin, clarithromycin, clindamycin, gentamicin, trimethoprim-sulfamethoxazole, and vancomycin were classified as broad-spectrum agents. This classification was used as an analytical framework for the present study and should not be interpreted as a validated antimicrobial-spectrum classification system. Because antimicrobial spectrum depends on the pathogen, infection site, and clinical context, these categories were treated as study-specific descriptive groupings rather than universal classifications of individual agents. Therapy was classified as empirical when treatment selection was not guided by microbiological identification and susceptibility information, whereas treatment was considered targeted when antibiotic selection or modification was supported by microbiological findings and, when available, antimicrobial susceptibility results. Targeted treatment therefore referred to documented pathogen-directed prescribing rather than simply the availability of a positive culture or antibiogram. Antibiotic treatment modification following microbiological results was evaluated separately from empirical or targeted treatment status, as described in Section 2.11. Patients who did not receive antibiotics were retained as a clinically defined category rather than being treated as having missing antibiotic exposure data. The individual antibiotic agents reported in the study represented antibiotics documented as administered during the index clinical episode. These records were not separately classified according to whether an agent was the initial, final, or principal antibiotic in the treatment sequence. Accordingly, the distribution of individual antibiotics was interpreted as documented treatment exposure rather than as a description of antibiotic sequencing.

### 2.8. Timing of Antibiotic Initiation and Pre-Antibiotic Microbiology

The timing of antibiotic initiation was evaluated as a distinct component of the therapeutic decision-making process. The relationship between the initiation of treatment and the clinical, laboratory, and microbiological information available at the time of the decision was examined using the information documented in the medical records. Antibiotic initiation varied according to the child’s clinical condition and the treating pediatrician’s assessment. In most cases, inflammatory laboratory results were available before antibiotic initiation; however, some measurements had been performed in primary healthcare settings approximately 6–12 h before hospital presentation and were subsequently used in clinical decision-making without repeat testing. Therefore, the timing of biomarker measurement did not necessarily coincide with the time of antibiotic initiation. Among patients undergoing microbiological testing, whether the specimen had been collected before antibiotic exposure was evaluated separately. The timing of specimen collection was distinguished from the availability of culture and antimicrobial susceptibility results, which were not necessarily available when empirical treatment was initiated. This information was used to characterize diagnostic stewardship and to assess the extent to which microbiological evidence contributed to the transition from empirical to targeted antimicrobial therapy. Because exact timestamps for the availability of individual laboratory and microbiological results were not systematically recorded in the analytical dataset, their availability at the initial treatment decision could not be verified for every patient. Consequently, analyses incorporating microbiological results obtained during the index episode were interpreted as retrospective associations rather than direct assessments of the information available to the treating pediatrician at the moment of initial antibiotic prescribing.

### 2.9. Bacterial Evidence Score

To summarize the cumulative evidence supporting a bacterial etiology, a study-specific Bacterial Evidence Score (BES) ranging from 0 to 6 points was constructed from six clinical, inflammatory, and microbiological indicators available in the analytical dataset. One point was assigned for each of the following findings: fever ≥ 38 °C, C-reactive protein (CRP) ≥ 40 mg/L, white blood cell count (WBC) ≥ 15 × 10^9^/L, absolute neutrophil count (ANC) ≥ 10 × 10^9^/L, procalcitonin (PCT) ≥ 0.5 ng/mL, and a positive bacterial culture. Findings below these thresholds, or absence of a positive culture, contributed zero points. For the microbiological component, both a documented negative culture and the absence of microbiological testing contributed zero points. However, these circumstances were retained as distinct categories in the dataset and were not considered clinically equivalent.

Individual component scores were summed to generate a total score ranging from 0 to 6, with progressively higher values representing a greater cumulative burden of evidence compatible with bacterial infection. Higher scores did not necessarily indicate a proportionally greater probability of confirmed bacterial infection. The selected thresholds were used to operationalize inflammatory and microbiological findings for exploratory analysis rather than to establish or confirm bacterial infection. Equal weighting of the six components was adopted as a pragmatic approach within the study-specific framework and did not imply equivalent diagnostic accuracy or clinical importance. Elevated inflammatory biomarkers are not specific to bacterial infection, while a positive culture must be interpreted in relation to the clinical presentation, specimen type, and possibility of colonization or contamination.

The score was analyzed primarily as an ordinal/continuous variable to preserve its graded structure, while individual score levels were also examined to assess changes in antibiotic prescribing across increasing levels of bacterial evidence. Associations estimated per one-point increase in BES were interpreted as summary associations, without assuming that each additional point represented an identical change in the probability of bacterial infection or antibiotic prescribing. The distribution of antibiotic prescribing across individual BES levels was therefore examined to identify potential non-linear or non-monotonic patterns that might not be captured by a single per-point association estimate.

The BES thresholds and the subsequent classification of antibiotic decision concordance represented operational choices within the study-specific retrospective analytical framework. Because prospective prespecification of these criteria could not be established from the available study documentation, they were not presented as a priori validated decision thresholds. The BES ≥ 2 cutoff was used to distinguish lower from higher cumulative evidence categories for exploratory comparisons, rather than to establish a clinically validated indication for antibiotic treatment. Accordingly, concordance with this threshold represented agreement with the study-defined analytical framework and not an independent determination of prescribing appropriateness. The sensitivity of the classification to the selected cutoff was examined using alternative BES thresholds. In addition, a five-component BES excluding the microbiological culture component was considered to assess the influence of culture availability on the study-defined classification.

The Bacterial Evidence Score was developed specifically as an analytical framework for the present retrospective study and should not be interpreted as a previously validated diagnostic or clinical prediction instrument. It was not intended to replace clinical judgment or to determine whether an individual pediatrician’s treatment decision was clinically appropriate. Furthermore, because the availability of individual microbiological results at the time of initial prescribing could not be established for every patient, the complete BES represented the evidence documented during the index clinical episode and did not necessarily reproduce the information available when the initial antibiotic decision was made.

### 2.10. Assessment of Antibiotic Decision Concordance

Concordance between the cumulative evidence supporting bacterial infection and the antibiotic treatment decision was assessed using a study-specific operational classification. Antibiotic decisions were classified as concordant or discordant according to the relationship between the Bacterial Evidence Score (BES) and whether antibiotic therapy was initiated or withheld. Decisions were considered concordant when antibiotic therapy was withheld in patients with limited bacterial evidence (Bacterial Evidence Score 0–1) or initiated in patients with stronger cumulative bacterial evidence (Bacterial Evidence Score ≥ 2). Conversely, decisions were classified as discordant when antibiotics were prescribed despite limited bacterial evidence (score 0–1) or withheld despite stronger evidence supporting bacterial infection (score ≥ 2).

The BES ≥ 2 threshold was applied as an exploratory operational cutoff within the study-specific analytical framework, rather than as a validated clinical threshold for initiating or withholding antibiotics. Consequently, the terms concordant and discordant describe agreement or disagreement with the predefined scoring-based classification used in the analysis and should not be interpreted as direct measures of guideline adherence, inappropriate prescribing, or individual clinical decision quality. Clinical circumstances not fully captured by the score, including infection severity, suspected infection site, comorbidities, reported drug allergies, and the treating pediatrician’s assessment, may have influenced treatment decisions.

This classification was based exclusively on information available for the analytical assessment of the treatment decision and did not incorporate subsequent clinical outcomes, thereby avoiding outcome-informed retrospective classification. However, because the complete BES incorporated microbiological information documented during the index episode, including culture results that may have become available after initial antibiotic initiation, the classification did not necessarily reflect only the evidence available at the exact time of the initial prescribing decision. This temporal limitation was considered when interpreting decision concordance.

The concordance framework was developed specifically for the present study as an antimicrobial stewardship-oriented analytical measure and should not be interpreted as a validated clinical decision rule. Decision discordance was defined as the primary outcome for the principal multivariable analysis. Because discordance was defined partly by the BES threshold itself, associations between BES and this outcome were considered structurally dependent and were not interpreted as independent evidence that higher bacterial evidence improves prescribing quality. Sensitivity analyses were performed using alternative six-component BES thresholds of ≥1 and ≥3, compared with the primary threshold of ≥2. For each threshold, discordance was recalculated as antibiotic prescribing below the specified cutoff or antibiotic withholding at or above that cutoff. An additional sensitivity analysis used a five-component BES excluding positive bacterial culture, with discordance classified at a threshold of ≥2. This analysis assessed the extent to which inclusion of microbiological results, which may not have been available at initial prescribing, influenced the operational classification. All sensitivity analyses retained the same analytical cohort and antibiotic treatment decisions, allowing differences in discordance estimates to be attributed to changes in the scoring framework rather than changes in the study population.

### 2.11. Microbiology-Guided Treatment Modification

The practical influence of microbiological information on antimicrobial management was assessed by examining the documented relationship between culture findings, antimicrobial susceptibility results, and subsequent treatment decisions. Among patients with positive cultures, the use of pathogen identification and, where available, antimicrobial susceptibility information to support targeted antibiotic selection or modification of an existing regimen was evaluated. Empirical therapy was defined as antibiotic treatment initiated without documented pathogen-specific microbiological guidance. Targeted therapy was defined as antibiotic selection or adjustment supported by an identified bacterial pathogen and relevant microbiological findings, including susceptibility results when available. Targeted therapy and treatment modification were evaluated as distinct variables because microbiologically guided treatment could involve either the selection of an antibiotic or the adjustment of an existing regimen. The availability of an antibiogram alone was not considered sufficient to classify treatment as targeted; documented use of microbiological information in antibiotic selection or adjustment was required. Treatment modification was defined as a documented change in the initial antibiotic regimen following the availability of microbiological findings, including substitution of the prescribed agent or another recorded microbiologically guided adjustment. A positive culture alone was not considered sufficient evidence of treatment modification. Culture–antibiotic alignment was assessed, where relevant information was available, by examining the relationship between the identified pathogen, documented susceptibility findings, and the recorded antibiotic regimen. This assessment was descriptive and was not treated as an independently validated measure of overall prescribing appropriateness. Patients with positive cultures who did not receive antibiotics during the index clinical episode were retained as a separate treatment category. In these cases, the available clinical documentation indicated reported antibiotic allergies or decisions to defer antibiotic initiation. Such cases were not automatically classified as inappropriate treatment, because a positive culture requires interpretation in the context of the patient’s clinical presentation and the possibility of colonization or contamination. The available data did not permit a standardized retrospective distinction between clinically significant bacterial infection, colonization, and specimen contamination in these cases. Consequently, the 44 culture-positive patients without antibiotic treatment were described as a separate subgroup rather than classified as confirmed cases of untreated bacterial infection. Because the exact timing of microbiological result availability was not systematically recorded for every patient, the analysis did not assume that culture or susceptibility results had been available when the initial antibiotic decision was made. Accordingly, microbiology-guided treatment modification was interpreted as a documented therapeutic response to microbiological information rather than evidence that the initial prescribing decision had been microbiologically informed.

### 2.12. Clinical Outcomes and Early Reassessment

Clinical outcome was classified into four categories: resolved/improved, partial improvement, no improvement, and clinical deterioration. Outcome classification was based on the treating pediatrician’s documented clinical assessment and the available laboratory findings during the index clinical episode. Resolved/improved represented documented resolution or clinically meaningful improvement in the patient’s condition, as assessed by the treating pediatrician. Partial improvement indicated improvement in some laboratory parameters without complete clinical improvement. No improvement indicated the absence of documented meaningful clinical recovery, whereas clinical deterioration represented documented worsening of the patient’s condition. These categories reflected routine clinical documentation rather than prospectively standardized outcome criteria. No independent clinical adjudication or retrospective application of standardized response criteria was performed. The four-category classification was retained for descriptive analyses. For binary outcome analyses, resolved/improved was classified as a favorable outcome, whereas partial improvement, no improvement, and clinical deterioration were combined as an unfavorable outcome. This binary grouping was used for analytical purposes and did not imply that partial improvement was clinically equivalent to treatment failure or deterioration. Accordingly, the unfavorable outcome category should not be interpreted as a direct measure of antibiotic treatment failure. Early reassessment was also recorded and evaluated as an indicator of documented clinical review following the initial therapeutic decision, including evaluation of treatment response and consideration of whether further management adjustments were required. Because the exact timing of reassessment was not consistently available as a structured variable, no fixed time threshold was retrospectively imposed for this measure. Clinical outcome assessments were performed at different time points according to the patient’s clinical course and the treating pediatrician’s documentation. Consequently, a uniform follow-up interval could not be established for the entire cohort, and the recorded outcomes should be interpreted as documented clinical status during the observed episode rather than standardized responses measured at a common time point. Similarly, the early reassessment variable indicated whether reassessment had been documented, rather than confirming that all patients had been reviewed within a prespecified number of hours. The absence of a documented reassessment was not assumed to indicate that clinical reassessment had not occurred. These limitations were considered when interpreting associations between BES-based decision concordance and clinical outcomes.

### 2.13. Primary and Secondary Outcomes

The primary study outcome was BES-based antibiotic decision discordance, defined as disagreement between the documented decision to prescribe or withhold antibiotics and the study-specific Bacterial Evidence Score (BES) threshold of ≥2. Specifically, discordance comprised antibiotic prescribing in patients with BES 0–1 and antibiotic withholding in patients with BES ≥ 2. This operational outcome was exploratory and did not constitute an independently validated measure of inappropriate prescribing or guideline nonadherence. Secondary outcomes included antibiotic prescribing, broad-spectrum versus narrow-spectrum therapy, empirical versus targeted treatment, antimicrobial susceptibility testing availability, microbiology-guided treatment modification, culture–antibiotic alignment, documented clinical outcome, and early reassessment. Clinical outcome was evaluated using the four documented categories and, for binary analyses, as favorable (resolved/improved) versus unfavorable (partial improvement, no improvement, or clinical deterioration). The association between the Bacterial Evidence Score and the probability of antibiotic prescribing was evaluated as a central secondary analysis of the prescribing gradient, while factors associated with BES-based decision discordance were examined in the principal multivariable model. Additional exploratory analyses assessed the relationship between decision discordance and unfavorable clinical outcome and the sensitivity of the discordance classification to alternative BES thresholds (≥1 and ≥3) and exclusion of the microbiological culture component. These analyses were intended to characterize the robustness and clinical interpretability of the study-specific classification rather than establish the diagnostic validity of the BES.

### 2.14. Statistical Analysis

Statistical analyses were performed using Python version 3.13.5. SciPy version 1.17.0 was used for inferential statistical testing, and statsmodels version 0.14.6 was used for multivariable regression analyses. Data management and analytical structuring were performed using pandas version 2.2.3. Categorical variables were summarized as absolute frequencies and percentages, whereas continuous variables included in the principal group comparisons were summarized as medians with interquartile ranges (IQRs). The Mann–Whitney U test was used to compare age, temperature, and continuous inflammatory biomarkers between patients who received antibiotics and those who did not. Pearson’s chi-square (χ^2^) test was used to compare categorical variables. The χ^2^ test was also applied to assess differences in antibiotic prescribing across primary diagnostic categories and across the 0–6 levels of the Bacterial Evidence Score (BES), as well as relevant associations between categorical microbiological and therapeutic variables. In a supplementary analysis, Pearson’s chi-square test was used to compare the distribution of the seven BES categories (scores 0–6) across primary clinical diagnoses. Diagnosis-specific BES values were summarized as means with standard deviations (SDs), and the number and proportion of patients with BES ≥ 2 were calculated for each diagnostic category (Supplementary Table S1). For this analysis, BES was recalculated directly from its six predefined binary components to ensure consistency with the operational scoring definition. Binary multivariable logistic regression was used to evaluate the principal binary outcomes. For the antibiotic-prescribing analysis, the Bacterial Evidence Score was modeled as an ordinal/continuous predictor, with its effect interpreted per one-point increase, after adjustment for age, sex, care setting, and primary diagnosis. Because the score represents a composite of indicators with potentially unequal clinical contributions, the estimated per-point association was interpreted as a model-based summary rather than evidence of a uniform effect across all score levels. To examine the individual contributions of the components underlying the composite score, an additional multivariable logistic regression model was fitted with antibiotic prescribing (yes/no) as the dependent variable. Fever ≥ 38 °C, CRP ≥ 40 mg/L, WBC ≥ 15 × 10^9^/L, ANC ≥ 10 × 10^9^/L, PCT ≥ 0.5 ng/mL, and positive bacterial culture were entered simultaneously as binary predictors, with adjustment for age, sex, care setting, and primary diagnosis. The model included 2688 patients, and results were expressed as adjusted odds ratios with 95% confidence intervals and *p*-values (Supplementary Table S2). These associations were interpreted as retrospective rather than causal, particularly because microbiological results may not have been available at the time of initial antibiotic prescribing and WBC and ANC may be correlated. For the principal analysis of decision discordance, discordance was specified as the dependent variable, and the multivariable model included age, sex, care setting, primary diagnosis, and calendar year. Because the Bacterial Evidence Score formed part of the operational definition used to classify decision concordance and discordance, it was not entered as an independent predictor in the discordance model, thereby avoiding structural circularity between outcome definition and predictor specification. A separate multivariable logistic regression model was used to evaluate the association between decision discordance and unfavorable clinical outcome, with adjustment for age, sex, care setting, primary diagnosis, calendar year, and Bacterial Evidence Score. This association was interpreted as observational and exploratory, given that the discordance classification was study-specific and that clinical outcomes were assessed at variable time points. Effect estimates were reported as adjusted odds ratios (aORs) with 95% confidence intervals (CIs) and *p*-values. Reference categories were specified for categorical predictors. Overall model significance was assessed using the likelihood-ratio test, and explanatory performance was summarized using McFadden’s pseudo-R^2^. Sensitivity analyses were conducted to assess the dependence of BES-based antibiotic decision discordance on the operational scoring threshold and inclusion of microbiological evidence. The primary six-component BES classification, using a threshold of ≥2, was compared with alternative thresholds of ≥1 and ≥3. For each threshold, discordance was recalculated using the same treatment-decision rule: antibiotic prescribing below the specified threshold or antibiotic withholding at or above that threshold. An additional analysis excluded the positive bacterial culture component, generating a five-component BES ranging from 0 to 5, with discordance defined using a threshold of ≥2. The number and proportion of discordant decisions were calculated for each specification using the same study population (*n* = 2688). These analyses were descriptive and exploratory; differences in discordance proportions were interpreted as sensitivity to the operational definition rather than evidence of diagnostic accuracy or clinical validation. All statistical tests were two-sided, and *p* < 0.05 was considered statistically significant. Statistical interpretation emphasized effect estimates and their 95% CIs in addition to *p*-values.

### 2.15. Data Management and Non-Applicable Categories

Before analysis, the internal consistency and logical structure of the 55-variable database have been checked. Situations in which a variable has not been clinically applicable have been distinguished from genuinely missing information. Accordingly, antibiotic-specific characteristics among patients who did not receive antibiotics and susceptibility-related variables among patients for whom the corresponding testing had not been performed have not been interpreted as conventional missing clinical data. Similarly, the absence of microbiological testing has not been classified as a negative culture, and the absence of antimicrobial susceptibility testing has not been interpreted as documented susceptibility or resistance. Analyses requiring microbiological testing, positive cultures, or susceptibility information have therefore been restricted to the corresponding eligible analytical populations.

### 2.16. Subgroup Analyses

Microbiology-specific analyses have been performed within the populations for whom the corresponding information has been available. Analyses of targeted therapy and microbiology-guided treatment modification have focused on patients with relevant positive microbiological findings, while the relationship between antimicrobial susceptibility testing and targeted treatment has been evaluated within the corresponding microbiologically evaluable population. The denominator for each subgroup analysis has been defined according to the population in whom the specific test, exposure, or treatment decision has been applicable, rather than automatically using the entire study cohort.

### 2.17. Ethical Considerations

This study has used a retrospective design based exclusively on existing data extracted from pediatric medical records. Data extraction has been performed using a structured data collection form developed for the study, which has included the predefined demographic, clinical, laboratory, microbiological, and pharmacotherapeutic variables specified in the study protocol. No prospective patient recruitment, direct patient contact, or intervention in diagnostic or therapeutic management has been undertaken.

The study protocol entitled “THE ROLE OF INFLAMMATORY BIOMARKERS AND MICROBIOLOGICAL PROFILE IN CLINICAL DECISION-MAKING FOR ANTIBIOTIC THERAPY IN PEDIATRIC PATIENTS: A RETROSPECTIVE STUDY” has been approved by the Ethics Committee of the General Hospital Gjilan, Department of Pediatrics, on 8 April 2026 (Approval No. 10/08/04/2026). Because of the retrospective nature of the study and the exclusive use of existing medical-record data, the requirement for individual informed consent has been waived in accordance with the Ethics Committee approval. Data have been anonymized during database preparation, and no directly identifying patient information has been included in the analytical dataset or reported in the manuscript. Study findings have been reported only in aggregate form.

## 3. Results

The final analytical cohort comprised 2688 pediatric patients evaluated between January 2023 and March 2026, with 55 predefined variables available in the analytical dataset. Antibiotic therapy was prescribed in 1974 patients (73.4%), whereas 714 (26.6%) were managed without antibiotics. Microbiological testing was obtained in 913 patients (34.0%), yielding 362 positive cultures; an antibiogram was available in 320 culture-positive cases. Among antibiotic-treated patients, 1807 (91.5%) received empirical therapy and 167 (8.5%) received targeted therapy, while 1391 (70.5%) received broad-spectrum agents. Based on the study-specific concordance algorithm, antibiotic decision-making was classified as concordant in 1863 patients (69.3%) and discordant in 825 (30.7%). Antibiotic prescribing generally increased across increasing levels of the Bacterial Evidence Score, from 36.9% at score 0 to 98.1% at score 6. Inflammatory biomarker levels, particularly CRP, procalcitonin, WBC, and ANC, were higher among antibiotic-treated children. Among culture-positive antibiotic-treated patients, targeted therapy was documented only in cases with an available antibiogram. Clinical resolution or improvement was documented in 2224 patients (82.7%), while 464 (17.3%) had partial improvement, no improvement, or clinical deterioration. Early reassessment was documented in 2101 patients (78.2%). Additional analyses demonstrated substantial variation in the recalculated Bacterial Evidence Score across primary diagnostic categories (Pearson’s χ^2^ = 2244.54, *p* < 0.001). The proportion of patients with BES ≥ 2 ranged from 1.7% among those diagnosed with viral upper respiratory infection to 100% among those with sepsis or suspected invasive infection (Supplementary Table S1). In a separate multivariable logistic regression model incorporating the six individual BES components and adjusting for age, sex, care setting, and primary diagnosis, CRP ≥ 40 mg/L (aOR 1.53, 95% CI 1.22–1.90), ANC ≥ 10 × 10^9^/L (aOR 1.65, 95% CI 1.22–2.24), PCT ≥ 0.5 ng/mL (aOR 1.54, 95% CI 1.20–1.99), and positive bacterial culture (aOR 1.46, 95% CI 1.03–2.07) were independently associated with antibiotic prescribing. Fever ≥ 38 °C and WBC ≥ 15 × 10^9^/L were not independently associated with prescribing after adjustment (Supplementary Table S2). These findings describe retrospective associations rather than validated diagnostic or causal relationships. Baseline demographic and clinical characteristics, together with inflammatory biomarker levels stratified by antibiotic prescribing status, are presented in Table 1.

Continuous variables are presented as median [IQR] and were compared using the Mann–Whitney U test; categorical variables were compared using Pearson’s chi-square test. Antibiotic-treated children showed substantially higher inflammatory activity, whereas age and sex distributions were similar between groups. The distribution of antibiotic prescribing and withholding across the nine primary clinical diagnostic categories is summarized in Table 2.

Diagnostic definitions: Urinary tract infection (UTI) referred to cases recorded with a clinical diagnosis of infection involving the urinary tract. Bacterial gastroenteritis referred to cases documented by the treating pediatrician as gastrointestinal infection of presumed or confirmed bacterial etiology. Acute bacterial sinusitis referred to cases documented with a clinical diagnosis of acute sinusitis considered bacterial in origin. These diagnostic categories were based on the medical records and were not independently reassessed using standardized diagnostic criteria or uniformly confirmed by microbiological testing. Antibiotic prescribing patterns across the seven levels of the study-specific Bacterial Evidence Score (0–6) are presented in Table 3.

The Bacterial Evidence Score (BES) was calculated by assigning one point to each of six study-specific indicators: fever ≥ 38 °C, CRP ≥ 40 mg/L, WBC ≥ 15 × 10^9^/L, ANC ≥ 10 × 10^9^/L, PCT ≥ 0.5 ng/mL, and positive bacterial culture. The total score ranged from 0 to 6. Percentages were calculated within each BES category. The BES was an exploratory, nonvalidated analytical measure and was not used to establish a confirmed bacterial diagnosis or determine clinical prescribing appropriateness.

Antibiotic prescribing differed significantly across Bacterial Evidence Score levels (Pearson’s chi-square *p* < 0.001). Although the pattern was not strictly monotonic at every adjacent score level, antibiotic use generally increased with greater cumulative bacterial evidence, from 36.9% at score 0 to 98.1% at score 6. In adjusted logistic regression, each one-point increase in the score was associated with higher odds of antibiotic prescribing (aOR 1.28, 95% CI 1.17–1.41; *p* < 0.001). The per-point estimate represents an overall model-based association and should not be interpreted as an identical increase in prescribing probability between every two adjacent BES categories. In particular, antibiotic prescribing was slightly lower at BES 4 (85.1%) than at BES 3 (86.2%), indicating that the observed relationship was not strictly monotonic. Because the score included microbiological results that may have become available after initial antibiotic initiation, these findings describe retrospective associations between documented bacterial evidence and prescribing rather than a validated prospective decision rule. The microbiological findings, identified bacterial pathogens, antimicrobial susceptibility testing availability, and documented resistance phenotypes are summarized in Table 4.

Microbiology was obtained in approximately one-third of the cohort, with 362 positive cultures. Susceptibility testing was available for 320 positive isolates (88.4% of positive cultures). MDR, ESBL, and MRSA phenotypes were uncommon but clinically relevant for treatment refinement. The distribution of individual antibiotic agents and the principal pharmacotherapeutic characteristics among antibiotic-treated patients are presented in Table 5.

Fourteen individual antibiotics were represented in the cohort. Empirical treatment predominated, and approximately seven in ten treated children received a broad-spectrum agent; most therapy was administered orally. Narrow-spectrum antibiotics included amoxicillin, ampicillin, penicillin V, and cephalexin. Broad-spectrum antibiotics included amoxicillin/clavulanate, cefuroxime, ceftriaxone, cefotaxime, azithromycin, clarithromycin, clindamycin, gentamicin, trimethoprim-sulfamethoxazole, and vancomycin, according to the study-specific operational classification. Empirical therapy was defined as antibiotic treatment initiated without documented pathogen-specific microbiological guidance, whereas targeted therapy referred to antibiotic selection or modification supported by pathogen identification and relevant microbiological findings, including susceptibility results when available. These spectrum categories were used for descriptive analysis and should not be interpreted as universally applicable classifications of individual agents. Individual antibiotic counts represent documented treatment exposure during the index clinical episode; the available records did not consistently distinguish initial, final, or principal antibiotics following treatment modification. Percentages were calculated among antibiotic-treated patients (*n* = 1974). Microbiology-guided antibiotic management, including targeted therapy, treatment modification, and the documented use of susceptibility information among culture-positive patients, is presented in Table 6.

Among culture-positive patients, 46.1% received targeted therapy and 25.1% underwent a documented treatment modification after microbiological results. Among culture-positive antibiotic-treated children (*n* = 318), targeted therapy was strongly associated with antibiogram availability (*p* < 0.001). The distribution of BES-based antibiotic decision concordance, documented clinical outcomes, and early clinical reassessment is summarized in Table 7.

Decision concordance was determined using the study-specific Bacterial Evidence Score (BES) threshold of ≥2. Concordant decisions included antibiotic withholding at BES 0–1 and antibiotic prescribing at BES ≥ 2. Discordant decisions included antibiotic prescribing at BES 0–1 or antibiotic withholding at BES ≥ 2. This exploratory classification was not independently validated and should not be interpreted as a measure of inappropriate antibiotic prescribing or guideline nonadherence. Clinical outcomes reflected the treating pediatrician’s documented assessment during the index episode rather than evaluation at a standardized follow-up time point. Percentages were calculated using the total cohort (*n* = 2688).

Using the study-specific concordance algorithm, 69.3% of antibiotic decisions were classified as concordant and 30.7% as discordant. Specifically, the discordant category comprised patients who received antibiotics despite BES 0–1 and those who did not receive antibiotics despite BES ≥ 2. These findings represent disagreement with the operational scoring rule rather than confirmed errors in clinical decision-making. More than four-fifths of patients had documented resolution or improvement. Partial improvement, no improvement, and clinical deterioration were recorded in 11.0%, 4.4%, and 1.9% of patients, respectively, while early reassessment was documented in 78.2%. Microbiology-guided treatment modification was documented in 91 patients (3.4% of the overall cohort). Unfavorable outcome did not differ significantly between concordant and discordant decisions in the unadjusted comparison (*p* = 0.249). In an adjusted analysis including age, sex, care setting, primary diagnosis, calendar year, and Bacterial Evidence Score, decision discordance was not independently associated with unfavorable outcome (aOR 0.88, 95% CI 0.69–1.13; *p* = 0.311). These associations should be interpreted cautiously because the BES-based classification was exploratory, microbiological information was not necessarily available at the initial prescribing decision, and clinical outcomes were not assessed at a uniform follow-up interval. The adjusted associations between demographic and clinical characteristics and BES-based antibiotic decision discordance, estimated using multivariable logistic regression, are presented in Table 8.

Adjusted odds ratios (aORs) were estimated using multivariable binary logistic regression, with discordant antibiotic decision-making as the dependent variable. Discordance was defined according to the study-specific BES ≥ 2 classification. The model included age, sex, care setting, primary diagnosis, and calendar year. Reference categories were female sex, emergency/outpatient care, acute bacterial sinusitis, and calendar year 2023. BES was not included as an independent predictor because it formed part of the operational definition of the dependent variable, thereby avoiding structural circularity. The classification was exploratory and should not be interpreted as an independently validated measure of inappropriate antibiotic prescribing. After adjustment for age, sex, care setting, primary diagnosis, and calendar year, lower odds of discordant antibiotic decision-making were observed for bacterial gastroenteritis (aOR 0.68, 95% CI 0.49–0.95; *p* = 0.023), sepsis or suspected invasive infection (aOR 0.06, 95% CI 0.03–0.11; *p* < 0.001), skin/soft-tissue infection (aOR 0.58, 95% CI 0.41–0.82; *p* = 0.002), and viral upper respiratory infection (aOR 0.34, 95% CI 0.24–0.49; *p* < 0.001), relative to acute bacterial sinusitis. Sex, care setting, age, calendar year, acute otitis media, community-acquired pneumonia, streptococcal pharyngotonsillitis, and urinary tract infection were not independently associated with discordance. The model was globally significant (likelihood-ratio *p* < 0.001; McFadden pseudo-R^2^ = 0.061). These findings indicate that agreement with the study-defined BES classification varied across diagnostic categories, independently of the demographic and clinical setting variables included in the model. However, the observed associations should not be interpreted as evidence that particular diagnoses independently determine the clinical appropriateness of antibiotic treatment. Because the outcome was defined using an exploratory BES threshold and microbiological information documented during the index episode, the results describe factors associated with retrospective classification rather than validated predictors of prescribing errors. The sensitivity of the study-specific antibiotic decision discordance classification to alternative BES thresholds and exclusion of the microbiological culture component is presented in Table 9.

BES, Bacterial Evidence Score. Discordance was defined as antibiotic prescribing below the specified BES threshold or withholding antibiotics at or above that threshold. The six-component score included fever, CRP, WBC, ANC, PCT, and positive bacterial culture; the five-component score excluded culture. All percentages use the full cohort (*n* = 2688) as the denominator. These are exploratory, study-specific classifications and do not establish inappropriate prescribing.

The proportion of decisions classified as discordant varied with the operational BES threshold, increasing from 22.7% (609/2688) at BES ≥ 1 to 45.8% (1231/2688) at BES ≥ 3, compared with 30.7% (825/2688) in the primary BES ≥ 2 analysis. Excluding the culture component while retaining the ≥2 threshold yielded a discordance proportion of 32.7% (880/2688). These findings demonstrate that the estimated frequency of discordance was sensitive to the scoring definition and should not be interpreted as a validated measure of inappropriate antibiotic prescribing. The culture-excluded analysis also reduced reliance on microbiological findings that might not have been available at the time of initial treatment selection.

## 4. Discussion

The present study provides a real-world assessment of how inflammatory biomarkers, microbiological evidence, and clinical characteristics have been translated into antibiotic treatment decisions in a large pediatric cohort. Several findings are particularly relevant from an antimicrobial stewardship perspective. Antibiotics were prescribed to 73.4% of the 2688 children, but prescribing varied markedly according to diagnosis and the cumulative strength of bacterial evidence. Antibiotic prescribing generally increased across increasing levels of the Bacterial Evidence Score, from 36.9% among children with a score of 0 to 98.1% among those with a score of 6. After adjustment for relevant clinical characteristics, each one-point increase in the score remained independently associated with higher odds of antibiotic initiation. Overall, 30.7% of antibiotic treatment decisions were classified as discordant according to the study-specific concordance algorithm, reflecting disagreement with the study-specific BES classification rather than independently confirmed inappropriate prescribing.

Together, these findings suggest that antibiotic decision-making was strongly responsive to accumulated evidence of bacterial infection, although important opportunities for further stewardship optimization remained. The overall antibiotic exposure observed in this cohort should be interpreted in the context of the heterogeneous diagnostic spectrum and inclusion of both children with relatively low probability of bacterial infection and those with conditions in which early antimicrobial treatment is clinically important. Previous multicenter pediatric evidence has shown that suboptimal antibiotic use remains common even in institutions with established antimicrobial stewardship activities. Tribble et al., in a national point-prevalence study across 32 United States children’s hospitals, reported that 25.9% of children receiving antibiotics for infectious indications received at least one suboptimal antibiotic. Common reasons for inappropriate use included bug–drug mismatch, prolonged surgical prophylaxis, overly broad empirical therapy, and unnecessary treatment, highlighting persistent opportunities for antimicrobial optimization [15]. Importantly, our findings similarly demonstrate that the evaluation of prescribing quality should extend beyond whether an antibiotic was prescribed and should consider whether the decision was supported by the available evidence and whether the selected spectrum was proportionate to the clinical situation. A particularly notable finding was the strong diagnostic variation in antibiotic prescribing. Antibiotics were prescribed to only 19.8% of children with viral upper respiratory infection, compared with 77–81% across several diagnoses commonly associated with bacterial disease and 96.0% among patients with sepsis or suspected invasive infection. The supplementary diagnosis-stratified analysis further demonstrated that the distribution of cumulative bacterial evidence varied substantially across clinical diagnoses, reinforcing the importance of interpreting the BES within the diagnostic context rather than applying a uniform threshold across all pediatric infections (Supplementary Table S1).

Such variation is clinically plausible and demonstrates that prescribing was not uniform across diagnostic categories. Nevertheless, antibiotic exposure among children ultimately classified with a viral phenotype remains an important antimicrobial stewardship concern. The European PERFORM study demonstrated substantial variation in empirical antibiotic prescribing across pediatric emergency settings and reported that 46.3% of febrile episodes assigned a final viral phenotype received empirical systemic antibiotics, with 80.3% of these receiving at least one antibiotic from the WHO Watch category [16]. This illustrates the persistent difficulty of making antibiotic decisions under diagnostic uncertainty and supports stewardship strategies that incorporate clinical probability, biomarkers, microbiological evidence, and subsequent reassessment rather than relying solely on the initial diagnostic label. One of the central findings of our study was the graded relationship between the Bacterial Evidence Score and antibiotic initiation. Although the pattern was not strictly monotonic at every adjacent score level, antibiotic prescribing generally increased with greater cumulative bacterial evidence, and each one-point increase in the score was independently associated with higher odds of antibiotic initiation (aOR 1.28, 95% CI 1.17–1.41; *p* < 0.001). This finding supports the concept that cumulative evidence may more closely reflect real-world clinical decision-making than isolated laboratory thresholds. Pediatric infectious disease assessment frequently requires integration of several imperfect diagnostic indicators, because individual inflammatory biomarkers cannot reliably distinguish bacterial from non-bacterial infection across all clinical contexts. In pediatric pneumonia, a systematic review and meta-analysis demonstrated that CRP and PCT performed better than WBC and ESR in differentiating bacterial from non-bacterial disease, although their diagnostic performance remained suboptimal, supporting the value of interpreting biomarker findings within a broader diagnostic framework [17]. The present results therefore support an evidence-integration approach; however, the study-specific Bacterial Evidence Score should not be interpreted as a validated clinical prediction rule. Its value in the present analysis is primarily to quantify the gradient of accumulated bacterial evidence and examine how that gradient corresponded with actual prescribing behavior. Recent evidence from adult critical care further illustrates the importance of integrating inflammatory biomarkers with clinical risk stratification. In a retrospective cohort of 120 critically ill patients with sepsis, Stoian et al. demonstrated that the neutrophil-to-lymphocyte ratio measured at 72 h was associated with mortality (AUC 0.765), while its combination with APACHE II improved prognostic discrimination (AUC 0.827). The Carmeli score, reflecting multidrug-resistance risk, also contributed to prognostic assessment [18]. Although these findings concern adult sepsis prognosis rather than pediatric antibiotic initiation, they reinforce the broader principle that inflammatory markers should be interpreted alongside clinical and microbiological risk indicators rather than as isolated determinants of therapeutic decisions.

The individual inflammatory markers also showed a consistent pattern. Children receiving antibiotics had higher CRP, WBC, ANC, PCT, ESR, and maximum temperature values than those who did not receive antibiotics, whereas age and sex did not meaningfully differentiate treatment groups. In the supplementary mutually adjusted analysis, CRP, ANC, PCT, and positive bacterial culture retained statistically significant associations with antibiotic prescribing, whereas fever and WBC did not, indicating that the individual components of the composite score were not equally associated with prescribing decisions (Supplementary Table S2).

These findings indicate that objective indicators of inflammatory activity contributed to therapeutic decision-making. Nevertheless, elevated inflammatory biomarkers should not automatically be interpreted as evidence of bacterial etiology or as an indication for antibiotic therapy. Recent prospective pediatric evidence has shown that PCT may reflect disease severity and treatment intensity rather than specific bacterial etiology. In children with infectious diarrhea, higher PCT levels were associated with greater antibiotic use and hospitalization, despite poor discrimination between Salmonella-positive and Salmonella-negative cases [19]. However, the effectiveness of biomarker-guided antibiotic strategies may depend on the clinical setting and the strength of existing antimicrobial stewardship practices. In the large multicenter BATCH randomized controlled trial involving 1949 hospitalized children, the addition of a PCT-guided algorithm did not reduce the duration of intravenous antibiotic therapy compared with usual care, while remaining non-inferior with respect to safety outcomes [20]. Thus, the most appropriate role of biomarkers may be to refine clinical probability and support initiation, continuation, or discontinuation decisions rather than to function as isolated prescribing triggers. The microbiological component of the present study provides an additional stewardship perspective. Microbiological testing was performed in approximately one-third of the cohort, and positive cultures were identified in 362 patients. Among positive cultures, antimicrobial susceptibility information was available for the majority of cases. However, only a subset of culture-positive patients received targeted therapy, and treatment modification following microbiological information occurred in approximately one-quarter of culture-positive cases. This distinction between obtaining diagnostic information and translating that information into therapeutic action is important. Diagnostic stewardship is effective only when actionable microbiological results are translated into therapeutic decisions. Reuter et al. demonstrated that implementation of rapid diagnostic testing in children with positive blood cultures reduced the time to optimal antimicrobial therapy and unnecessary antibiotic exposure without worsening clinical outcomes [21]. The importance of translating microbiological findings into timely antimicrobial optimization is further supported by a recent retrospective study of 234 adult intensive care episodes involving Acinetobacter species. Stoian et al. reported that empirical antibiotics were initiated within 24 h in 95.7% of patients, but only 49.6% received at least one empirical agent subsequently confirmed to be microbiologically active. Moreover, active treatment within 72 h was documented in only 8.5% of cases, while early inflammatory biomarkers showed limited discriminatory value for carbapenem susceptibility [22]. Although these findings arise from a substantially different clinical population, they emphasize that early antibiotic initiation, microbiological susceptibility, and timely adjustment to active therapy represent distinct components of antimicrobial stewardship. This distinction is relevant to our findings, in which microbiological testing, targeted treatment, and subsequent treatment modification were documented at different frequencies.

These findings reinforce the principle that the clinical value of microbiological testing depends not merely on test availability but on the speed and consistency with which results influence antimicrobial management. Conversely, diagnostic technology alone may not guarantee improved prescribing. An Australian pediatric study evaluating rapid diagnostic methods for bacteremia found no significant improvement in effective or optimal antibiotic therapy when rapid diagnostics were introduced in isolation, leading the authors to emphasize the need to combine diagnostic information with active real-time antimicrobial stewardship and treatment modification [23]. This observation is highly relevant to our results. Although antibiogram availability was strongly associated with targeted treatment among microbiologically evaluable patients, the relatively limited frequency of documented treatment modification indicates that additional opportunities may exist to strengthen the transition from microbiological diagnosis to antimicrobial action. The finding should nevertheless be interpreted cautiously because targeted therapy and antibiogram availability are conceptually related; therefore, this association primarily reflects the clinical pathway from susceptibility information to directed therapy rather than an independent causal effect. An additional finding requiring cautious interpretation was the subgroup of 44 patients with positive bacterial cultures who did not receive antibiotic treatment. A positive culture does not necessarily establish an indication for antimicrobial therapy, particularly when the clinical significance of the isolated organism, specimen characteristics, potential colonization or contamination, and individual patient circumstances are considered. Accordingly, these cases should not be interpreted as confirmed instances of untreated bacterial infection or inappropriate antibiotic withholding. The inability to establish precisely when microbiological results became available further limits retrospective assessment of the initial prescribing decision.

The importance of diagnostic stewardship is also supported by evidence showing that more testing is not necessarily synonymous with better stewardship. In the multicenter Bright STAR Collaborative, implementation of blood-culture diagnostic stewardship across pediatric intensive care units was associated with a 33% reduction in blood culture rates and a 13% reduction in broad-spectrum antibiotic use, without adverse changes in mortality, readmission, length of stay, sepsis, or severe sepsis/septic shock [24]. The implication for our findings is that the objective should not be indiscriminate expansion of microbiological testing, but appropriate pre-antibiotic specimen collection in patients with sufficient clinical indication, followed by timely interpretation and therapeutic response. This distinction is particularly relevant because microbiological testing was not performed in all patients in our cohort and the absence of testing cannot be interpreted as a negative microbiological result. Broad-spectrum antibiotic exposure represents another important stewardship signal in the present study. Among antibiotic-treated children, 70.5% received agents classified as broad-spectrum, whereas 29.5% received narrow-spectrum treatment. The predominance of broad-spectrum therapy warrants attention, although it should not automatically be interpreted as inappropriate because spectrum requirements differ substantially according to diagnosis, disease severity, resistance risk, and care setting. This finding reinforces the importance of diagnosis-specific stewardship rather than applying a uniform spectrum-reduction strategy across all pediatric infections. Evidence from pediatric primary care has shown that multifaceted stewardship interventions can substantially reduce prescriptions for broad-spectrum agents, including amoxicillin-clavulanate and third-generation cephalosporins [25]. Similarly, implementation of an antimicrobial stewardship program in pediatric urgent-care settings improved guideline-concordant antibiotic selection, dosing, and duration, demonstrating that prescribing quality is multidimensional and extends beyond the decision of whether to prescribe an antibiotic [26]. The clinical relevance of stewardship interventions must ultimately be considered alongside patient outcomes. In the present cohort, 82.7% of patients had documented resolution or improvement, while 17.3% experienced partial improvement, no improvement, or clinical deterioration. Notably, decision concordance was not significantly associated with unfavorable clinical outcome in either the unadjusted analysis or the adjusted model. This negative result is important and should not be interpreted as evidence that concordant prescribing lacks clinical value. Clinical outcomes in pediatric infections are influenced by numerous factors beyond the initial antibiotic decision, including underlying diagnosis, disease severity, natural disease course, supportive care, treatment adherence, microbiological susceptibility, and subsequent therapeutic modification. Moreover, many uncomplicated infections resolve independently of antibiotic exposure. Consequently, a stewardship measure designed to assess the appropriateness of an initial treatment decision should not necessarily be expected to independently predict short-term clinical outcome. This interpretation is consistent with evidence that stewardship interventions can reduce antimicrobial exposure without compromising patient safety. Long-term implementation of a pediatric antimicrobial stewardship program based on guideline development and prospective audit with feedback was associated with substantial reductions in overall and broad-spectrum antibiotic use without changes in sepsis-related mortality [27]. Likewise, a randomized trial of clinical decision support for pediatric pneumonia demonstrated that its effectiveness varied according to care disposition: guideline-concordant antibiotic prescribing improved among children discharged from the emergency department but not among hospitalized patients, with no differences in delayed intensive care or revisit-related safety outcomes [28]. These findings are relevant to our observation that stewardship quality and clinical outcome should be regarded as complementary rather than interchangeable endpoints. Decision discordance was identified in 30.7% of the cohort. In the adjusted model, bacterial gastroenteritis, sepsis or suspected invasive infection, skin/soft-tissue infection, and viral upper respiratory infection were independently associated with lower odds of discordant decision-making compared with acute bacterial sinusitis, whereas age, sex, care setting, calendar year, acute otitis media, community-acquired pneumonia, streptococcal pharyngotonsillitis, and urinary tract infection were not independently associated with discordance. These differences suggest that discordance may arise more frequently in clinical conditions characterized by greater diagnostic ambiguity or variation in treatment thresholds rather than from uniform prescribing behavior across pediatric infections. Previous multicenter research has similarly demonstrated substantial variation in the appropriateness of pediatric antibiotic prescribing across clinical services, supporting the need for stewardship interventions tailored to specific prescribing contexts [29]. Diagnosis-specific feedback and stewardship algorithms may therefore be more effective than interventions based exclusively on overall prescribing rates. The sensitivity analyses further demonstrated that the estimated frequency of antibiotic decision discordance depended substantially on the operational definition of bacterial evidence. Discordance was observed in 22.7% of patients using a six-component BES threshold of ≥1, 30.7% using the primary threshold of ≥2, and 45.8% using a threshold of ≥3. Excluding the microbiological culture component while retaining the ≥2 threshold yielded a discordance proportion of 32.7%. These differences indicate that the observed discordance frequency is sensitive to both the selected cutoff and the composition of the score. Importantly, the primary estimate of 30.7% should not be interpreted as the prevalence of clinically inappropriate antibiotic prescribing, because the BES was neither prospectively validated nor independently adjudicated against established clinical prescribing criteria. The culture-excluded analysis also highlights the importance of distinguishing information documented during the clinical episode from evidence available at the moment of initial treatment selection. Accordingly, the BES-based classification may be useful for exploratory stewardship assessment, but its clinical relevance and optimal thresholds require prospective evaluation.

The finding that early reassessment was documented in 78.2% of patients is encouraging because reassessment represents a critical opportunity to revise empirical decisions once the clinical trajectory and additional diagnostic information become available. The initial antibiotic decision should not be viewed as a fixed endpoint. Instead, antimicrobial stewardship is a dynamic process that includes reassessment of indication, spectrum, route, duration, microbiological results, and clinical response. Structured and automated approaches to evaluating guideline concordance have increasingly been investigated in pediatrics, including algorithms that use structured and unstructured electronic health-record data to classify antibiotic selection as guideline-concordant or potentially discordant [30]. In this context, the integrated structure used in our study—combining bacterial evidence, treatment decision, microbiological refinement, concordance, and subsequent outcome—may provide a useful framework for future prospective stewardship evaluation. Taken together, our findings indicate that pediatric antibiotic prescribing in this cohort was strongly related to the cumulative strength of evidence supporting bacterial infection, but important stewardship opportunities remained in reducing decision discordance, optimizing broad-spectrum use, strengthening microbiology-guided treatment refinement, and ensuring systematic reassessment. The principal contribution of this study lies not in proposing a single biomarker or score as a replacement for clinical judgment, but in demonstrating how multiple layers of evidence can be examined together within real-world pediatric antibiotic decision-making. Future prospective multicenter studies should determine whether structured integration of clinical findings, inflammatory biomarkers, and timely microbiological information can improve concordance and reduce unnecessary antimicrobial exposure while maintaining favorable clinical outcomes.

### Strengths and Limitations

This study has several strengths. The relatively large cohort of 2688 pediatric patients and the inclusion of data collected over more than three years have enabled a comprehensive assessment of antibiotic decision-making across different clinical diagnoses. The integration of clinical characteristics, inflammatory biomarkers, microbiological findings, antimicrobial susceptibility data, resistance patterns, and pharmacotherapeutic variables has allowed antibiotic use to be evaluated as a multistep decision-making process rather than as an isolated prescribing event. Furthermore, the assessment of antibiotic initiation, antimicrobial spectrum, empirical versus targeted therapy, microbiology-guided treatment modification, decision concordance, clinical outcomes, and early reassessment provides a broad antimicrobial and diagnostic stewardship perspective. Several limitations should be acknowledged. The retrospective, single-center design limits causal inference and may affect the generalizability of the findings to other pediatric settings. Because data were extracted from routinely documented medical records, the analyses depended on the completeness and accuracy of the available documentation. Microbiological testing was performed according to clinical indication rather than systematically across the entire cohort, which may have introduced selection bias and restricted microbiology-related analyses to eligible subgroups. The Bacterial Evidence Score is a study-specific analytical construct and has not been externally validated; therefore, it should not be interpreted as a clinical prediction tool or diagnostic threshold. Similarly, decision concordance represents a study-specific stewardship-oriented analytical measure rather than an independent gold standard of prescribing appropriateness. The sensitivity analyses demonstrated that the estimated discordance proportion varied according to the selected BES threshold and inclusion of the microbiological component, emphasizing the operational rather than validated nature of the primary classification. In addition, exact microbiological result-availability times were not consistently documented, and clinical outcomes were assessed at nonuniform follow-up intervals. These limitations prevent the BES-based classification from being interpreted as a prospective prescribing decision rule and restrict conclusions about its relationship with subsequent clinical outcomes.

Residual confounding from unmeasured clinical factors cannot be excluded, and clinical outcomes were based on documented follow-up rather than prospective standardized assessment. Nevertheless, the integration of clinical, biomarker, microbiological, and pharmacotherapeutic information provides a detailed real-world evaluation of pediatric antibiotic decision-making.

## 5. Conclusions

Antibiotic decision-making in this pediatric cohort was strongly associated with the cumulative evidence supporting bacterial infection. Antibiotic prescribing generally increased across higher levels of the Bacterial Evidence Score, while substantial variation was observed across clinical diagnoses. The estimated frequency of BES-based decision discordance was sensitive to the selected scoring threshold and microbiological component, underscoring that this exploratory classification does not constitute an independently validated measure of inappropriate antibiotic prescribing.

Decision discordance remained common and varied according to diagnostic category, highlighting opportunities for diagnosis-specific antimicrobial stewardship. Despite generally favorable clinical outcomes, relevant stewardship opportunities remained, particularly regarding discordant treatment decisions, broad-spectrum antibiotic use, and the translation of microbiological and susceptibility results into targeted therapy. These findings support an integrated approach in which clinical assessment, inflammatory biomarkers, and microbiological evidence are interpreted collectively rather than in isolation. Strengthening diagnostic stewardship, timely reassessment, and microbiology-guided treatment refinement may contribute to more precise antibiotic use in pediatric practice while preserving favorable clinical outcomes.

## Figures and Tables

**Table 1:** Demographic, clinical, and inflammatory characteristics according to antibiotic prescribing.

Characteristic	Antibiotic (*n* = 1974)	No Antibiotic (*n* = 714)	*p*-Value
Age, years	8.8 [4.6–13.3]	8.9 [4.4–13.1]	0.617
Female sex, *n* (%)	981 (49.7)	364 (51.0)	0.586
Inpatient setting, *n* (%)	645 (32.7)	92 (12.9)	<0.001
Temperature, °C	38.1 [37.6–38.6]	37.7 [37.2–38.2]	<0.001
Fever ≥ 38 °C, *n* (%)	1126 (57.0)	279 (39.1)	<0.001
CRP, mg/L	59.0 [31.4–94.4]	25.4 [12.1–55.6]	<0.001
WBC, ×10^9^/L	13.0 [10.4–15.4]	10.7 [8.1–13.2]	<0.001
ANC, ×10^9^/L	8.6 [6.7–10.7]	6.4 [4.2–8.6]	<0.001
Procalcitonin, ng/mL	0.4 [0.2–0.8]	0.1 [0.1–0.3]	<0.001
ESR, mm/h	29.0 [19.0–39.0]	25.0 [12.0–36.0]	<0.001

**Table 2:** Antibiotic prescribing according to primary clinical diagnosis.

Primary Diagnosis	Total, *n*	Antibiotic, *n* (%)	No Antibiotic, *n* (%)
Viral upper respiratory infection	343	68 (19.8)	275 (80.2)
Acute otitis media	307	238 (77.5)	69 (22.5)
Urinary tract infection	305	239 (78.4)	66 (21.6)
Bacterial gastroenteritis	303	240 (79.2)	63 (20.8)
Sepsis/suspected invasive infection	299	287 (96.0)	12 (4.0)
Streptococcal pharyngotonsillitis	293	233 (79.5)	60 (20.5)
Community-acquired pneumonia	289	234 (81.0)	55 (19.0)
Acute bacterial sinusitis	285	220 (77.2)	65 (22.8)
Skin/soft-tissue infection	264	215 (81.4)	49 (18.6)

Antibiotic prescribing differed significantly across diagnostic groups (Pearson’s chi-square *p* < 0.001). Use was lowest for viral upper respiratory infection (19.8%) and highest for sepsis/suspected invasive infection (96.0%), demonstrating strong diagnosis-dependent treatment selection.

**Table 3:** Antibiotic prescribing across the bacterial evidence score.

Bacterial Evidence Score	Total, *n*	Antibiotic, *n* (%)	No Antibiotic, *n* (%)
0	401	148 (36.9)	253 (63.1)
1	626	421 (67.3)	205 (32.7)
2	680	543 (79.9)	137 (20.1)
3	499	430 (86.2)	69 (13.8)
4	268	228 (85.1)	40 (14.9)
5	162	153 (94.4)	9 (5.6)
6	52	51 (98.1)	1 (1.9)

**Table 4:** Microbiological profile, susceptibility testing, and resistance phenotypes.

Microbiological Characteristic	*n* (%) of Total Cohort
Microbiology ordered	913 (34.0)
Positive culture	362 (13.5)
Negative culture	551 (20.5)
Antibiogram available	320 (11.9)
MDR phenotype	43 (1.6)
ESBL phenotype	14 (0.5)
MRSA phenotype	4 (0.1)
Klebsiella pneumoniae	46 (1.7)
Staphylococcus aureus	38 (1.4)
Haemophilus influenzae	35 (1.3)
Escherichia coli	34 (1.3)
Salmonella spp.	33 (1.2)
Campylobacter jejuni	32 (1.2)
Proteus mirabilis	30 (1.1)
Enterococcus faecalis	30 (1.1)
Streptococcus pyogenes	29 (1.1)
Streptococcus pneumoniae	28 (1.0)
Moraxella catarrhalis	27 (1.0)

**Table 5:** Antibiotic agents and principal treatment characteristics among treated patients.

Treatment Characteristic	*n*	% of Antibiotic-Treated
Amoxicillin	155	7.9
Vancomycin	153	7.8
Amoxicillin-clavulanate	152	7.7
Ampicillin	152	7.7
Azithromycin	148	7.5
Cefotaxime	146	7.4
Clarithromycin	142	7.2
Trimethoprim-sulfamethoxazole	142	7.2
Penicillin V	139	7.0
Cephalexin	137	6.9
Cefuroxime	131	6.6
Clindamycin	131	6.6
Gentamicin	124	6.3
Ceftriaxone	122	6.2
Broad-spectrum therapy	1391	70.5
Narrow-spectrum therapy	583	29.5
Empirical therapy	1807	91.5
Targeted therapy	167	8.5
Oral route	1544	78.2
Intravenous route	430	21.8

**Table 6:** Microbiology-guided antibiotic management among culture-positive patients.

Management Indicator	*n*	% of Culture-Positive
Culture-positive patients	362	100.0
Antibiotic-treated after positive culture	318	87.8
Targeted therapy	167	46.1
Empirical therapy despite positive culture	151	41.7
No antibiotic despite positive culture	44	12.2
Therapy modified after microbiology	91	25.1
Antibiogram available	320	88.4

**Table 7:** Decision concordance, clinical outcomes, and reassessment.

Outcome/Management Measure	*n*	%
Concordant decision	1863	69.3
Discordant decision	825	30.7
Resolved/improved	2224	82.7
Partial improvement	297	11.0
No improvement	117	4.4
Clinical deterioration	50	1.9
Early reassessment documented	2101	78.2
Therapy changed after microbiology	91	3.4

**Table 8:** Multivariable logistic regression for determinants of discordant antibiotic decision-making.

Predictor	Adjusted OR	95% CI	*p*-Value
Male sex	0.98	0.83–1.16	0.838
Inpatient setting	0.87	0.71–1.07	0.196
Acute otitis media	0.72	0.52–1.00	0.051
Bacterial gastroenteritis	0.68	0.49–0.95	0.023
Community-acquired pneumonia	0.73	0.52–1.03	0.071
Sepsis/suspected invasive infection	0.06	0.03–0.11	<0.001
Skin/soft-tissue infection	0.58	0.41–0.82	0.002
Streptococcal pharyngotonsillitis	0.75	0.54–1.05	0.093
Urinary tract infection	0.75	0.54–1.04	0.085
Viral upper respiratory infection	0.34	0.24–0.49	<0.001
Calendar year 2024	0.94	0.76–1.17	0.609
Calendar year 2025	0.93	0.75–1.15	0.480
Calendar year 2026	0.74	0.51–1.07	0.105
Age (per year)	0.99	0.97–1.00	0.107

**Table 9:** Sensitivity analyses of BES-based antibiotic decision discordance.

BES-Based Classification	Discordant Decisions, *n*	Discordant Decisions, %
Six-component BES ≥ 1	609	22.7
Six-component BES ≥ 2 (primary analysis)	825	30.7
Six-component BES ≥ 3	1231	45.8
Five-component BES ≥ 2 (culture excluded)	880	32.7

## Data Availability

The data analyzed in this study were obtained from retrospective pediatric medical records and contain clinical information subject to institutional confidentiality and data-protection requirements. The dataset is therefore not publicly available. Anonymized data may be made available by the corresponding author upon reasonable request and subject to approval by the General Hospital Gjilan and applicable ethical and institutional requirements.

## Supplementary Materials

The following supporting information can be downloaded at: https://www.mdpi.com/article/10.3390/antibiotics15101020/s1, Table S1: Distribution of the recalculated Bacterial Evidence Score by primary clinical diagnosis; Table S2: Individual BES components associated with antibiotic prescribing in multivariable logistic regression.
