# Supplementary material for: The Role of Inflammatory Biomarkers and Microbiological Profile in Clinical Decision-Making for Antibiotic Therapy in Pediatric Patients: A Retrospective Study
Source: Antibiotics (Basel). 2026 Oct 14;15(10):1020. doi: 10.3390/antibiotics15101020 (PMC13700592; doi:10.3390/antibiotics15101020)
Supplement: Supplementary file 1 [file antibiotics-15-01020-s001.zip › antibiotics-4597737-supplementary.pdf]

SUPPLEMENTARY MATERIAL

The Role of Inflammatory Biomarkers and Microbiological Profile in Clinical Decision-Making for Antibiotic Therapy in Pediatric Patients: A Retrospective Study

Supplementary Table S1. Distribution of the recalculated Bacterial Evidence Score by primary clinical diagnosis

Primary clinical diagnosis	Patients, n	BES, mean (SD)	BES ≥2, n (%)
Viral upper respiratory infection	343	0.25 (0.49)	6 (1.7)
Acute otitis media	307	2.11 (1.20)	205 (66.8)
Urinary tract infection	305	1.98 (1.27)	188 (61.6)
Bacterial gastroenteritis	303	2.11 (1.17)	204 (67.3)
Sepsis / suspected invasive infection	299	4.37 (1.10)	299 (100.0)
Streptococcal pharyngotonsillitis	293	2.12 (1.18)	195 (66.6)
Community-acquired pneumonia	289	2.01 (1.14)	184 (63.7)
Acute bacterial sinusitis	285	2.18 (1.17)	194 (68.1)
Skin/soft-tissue infection	264	2.16 (1.21)	186 (70.5)
Total	2688	2.11 (1.50)	1661 (61.8)

BES was recalculated from six binary components: fever ≥38 °C, CRP ≥40 mg/L, WBC ≥15 × 10^9/L, ANC ≥10 × 10^9/L, PCT ≥0.5 ng/mL, and a positive bacterial culture (one point each; range 0–6). Positive culture contributed zero points if testing was negative or not performed; these two categories remained distinct in the source dataset. The distribution across the seven BES levels differed by primary diagnosis (Pearson $\chi^2 = 2244.54$, df = 48, $p < 0.001$). The BES is an exploratory, nonvalidated score and does not establish bacterial infection or prescribing appropriateness.

Supplementary Table S2. Individual BES components associated with antibiotic prescribing in multivariable logistic regression

Component	Adjusted OR	95% CI	p-value
Fever ≥38 °C	1.14	0.93–1.41	0.205
CRP ≥40 mg/L	1.53	1.22–1.90	<0.001
WBC ≥15 × 10^9/L	0.79	0.58–1.07	0.131
ANC ≥10 × 10^9/L	1.65	1.22–2.24	0.001
PCT ≥0.5 ng/mL	1.54	1.20–1.99	<0.001
Positive bacterial culture	1.46	1.03–2.07	0.031

Outcome: any antibiotic prescribed during the index clinical episode (yes/no). All six binary BES components were entered simultaneously, with adjustment for age (years), sex, care setting, and primary diagnosis (categorical). Analysis included 2,688 patients. McFadden pseudo-$R^2 = 0.226$. OR, odds ratio; CI, confidence interval; CRP, C-reactive protein; WBC, white blood cell count; ANC, absolute neutrophil count; PCT, procalcitonin. The microbiological component may reflect information obtained after initial prescribing; associations are retrospective and should not be interpreted causally. Correlation between WBC and ANC may influence their mutually adjusted estimates.

Analytical provenance

Source: PEDIATRIC ANTIBIOTICS FINALE(1).xlsx (2,688 patient records; 55 original variables). The score was independently recalculated from the original component variables, without overwriting the existing bacterial_evidence_score or decision_concordance columns. The recalculated BES distribution and BES ≥2 discordance count (825) match the main manuscript. Statistical analysis: Python 3 with pandas, SciPy (Pearson chi-square), and statsmodels (maximum-likelihood logistic regression); two-sided tests. These supplementary results describe a secondary analysis of the source dataset.
